# The Influence of Chronic and Situational Social Status on Stereotype Susceptibility

**DOI:** 10.1371/journal.pone.0144582

**Published:** 2015-12-08

**Authors:** Vincent Pillaud, David Rigaud, Alain Clémence

**Affiliations:** Institute of Psychology, University of Lausanne, Lausanne, Switzerland; University of Tuebingen Medical School, GERMANY

## Abstract

We tested whether stereotypical situations would affect low-status group members' performance more strongly than high-status group members'. Experiment 1 and 2 tested this hypothesis using gender as a proxy of chronic social status and a gender-neutral task that has been randomly presented to favor boys (men superiority condition), favor girls (women superiority condition), or show no gender preference (control condition). Both experiments found that women’s (Experiment 1) and girls’ performance (Experiment 2) suffered more from the evoked stereotypes than did men's and boys’ ones. This result was replicated in Experiment 3, indicating that short men (low-status group) were more affected compared to tall men (high-status group). Additionally, men were more affected compared to women when they perceived height as a threat. Hence, individuals are more or less vulnerable to identity threats as a function of the chronic social status at play; enjoying a high status provides protection and endorsing a low one weakens individual performance in stereotypical situations.

## Introduction

Literature on stereotype threat suggests that stereotypes could affect all of us [1]. This effect, originally reported for Black individuals [2], has rapidly extended to nearly all social groups. To name a few, stereotype threat affects women and children, as well as White men, people from low-economic status, and the elderly [3]. However, although most studies confirmed the negative influence of stereotype threat on stigmatized group members, only a few have tested its effects on individuals belonging to high-status groups (see [4], [5] for example). The present research was designed to investigate the role of social status in performance by postulating that stereotypes should affect low-status individuals more compared to high-status individuals.

Despite recent efforts to reduce as much as possible the nefarious effects of stereotypes and to promote equal opportunities between minorities and people of higher status groups, important inequalities remain in the workforce [6]. Much research has been devoted to better understanding factors that contribute to such unbalanced repartition and the role of stereotypes has been amongst the most vividly debated potential force at play. Indeed, some scholars have proposed that stereotypes could be used to legitimize and maintain the hierarchical organization of society [7], [8]. The persistence of status asymmetry in society would thus be served by values and stereotypes that still discriminate against members of low-status groups [9], [10]. Yet, even if the role of stereotype threat in performance is now widely acknowledged, research has shown that it does not only impact the performance of low-status individuals as we could have imagined [5] but that high-status individuals (such as men) can also suffer from it. How can we reconcile these two apparently contradictory findings?

### Social status and threatening situations

Leyens, Désert, Croizet and Darcis presented an enriched and informed discussion on generalizing the stereotype threat effects to high-status group members [5]. These authors claimed that if a dominant group “could experience stereotype threat, then it is very unlikely that chronic lower status or a history of stigmatization are preconditions of stereotype threat” (p. 1190). To reach such a conclusion, the researchers asked their male participants, that is, the members of a high-status group, to complete different tasks containing affective information (i.e., a negative stereotype for men) or making no reference to affect (a control condition). They observed that male participants, but not female ones, performed less well when affect was made salient.

Although this article demonstrated the presence of stereotype threat among men, it is unclear whether it has really tested if stereotyped situations affect high-status and low-status individuals the same way. As the authors stated, the stereotype threat situation did not affect the historical asymmetry between men and women in their study (i.e., the chronic status acquired through socialization processes) and men were still viewed as part of the dominant group. Instead, their manipulation affected a subordinate level of the intergroup comparison, that is, the affective domain, which, consequently, primed specific situational statuses. More precisely, women were presented as better than were men, attributing a low situational status to men (and a high one to women). A study by Stone, Lynch, Sjomeling, and Darley provides support for the importance of accounting for both situational and chronic statuses when investigating stereotype threat [11]. In this study, both White and Black participants were asked to engage in a simulated golf game. Participants were told either that the study focused on athletic ability (a positive stereotype for Black participants) or that it focused on the strategic intelligence in sports domain (a negative stereotype for Black participants). In the third condition, they were either primed (prime condition) or not primed (no-prime condition) for race prior to performing. The results were compared to a control condition that showed no difference in performance between Black and White participants. As hypothesized, the African American participants underperformed in the strategic intelligence and prime conditions while the European American participants obtained worse score in the athletic ability condition. It was found that Black participants underperformed White participants in both the prime condition (i.e., when their chronic low status was activated) and the strategic intelligence condition (i.e., when they endorsed a low situational status) whereas they outperformed White participants in the athletic ability condition (i.e., when they endorsed a high situational status). In both studies, the chronic status was overridden in an experimental situation by a stereotype that led the low-status individuals to perform better (as in [11]) or the high-status individuals to perform less well (as in [5]). In other words, situational status, rather than chronic status, yielded greater stereotype susceptibility in both groups. A recent study by Martiny, Roth, Jelenec, Steffens & Croizet also revealed that situational status could lead to stereotype threat effects by itself in newly formed groups [12]. However, if anyone can be affected by stereotypes, research also suggests that individuals could react differently in threatening situations as a function of their chronic statuses.

### Group-status and susceptibility to stereotypes

Research on stereotype threat has originally been conducted to explain the reasons that lead stigmatized groups to underperform when they face an evaluative situation [13]. Indeed, whereas they perform equally well in neutral situations, the performance gap between these groups increases when they believe that the task measures their competence. Stereotypes have logically been discussed as one of the factors that maintain group inequalities as mentioned before (such as the gender gap in mathematics for instance [14], [15]). However, most research has focused on individual differences that can amplify or narrow the negative effect of stereotypes (see Inzlicht, Aronson and Mendoza-Denton for a review, [16]) and only a few group comparisons have provided insights into whether endorsing a low-status identity can increase stereotype susceptibility.

Nonetheless, in a recent study, Pavlova, Weber, Simoes and Sokolov [17] invoked gender stereotypes and asked the male and female participants to perform a gender-neutral task in the face of gender stereotypes. They found that women were more affected by stereotypes compared to men whereas both men and women performed similarly in a control condition. These recent findings could be integrated in the more general perspective that we propose in this paper. We argue that stereotype threat effects could originate from two different contingent factors: it could both be observed on individuals endorsing a low chronic status in a typical stereotype threat situation and / or on individuals confronted with a low situational status. We consequently suggest that stereotypes should lead individuals with a low chronic status to being more susceptible to stereotype threat than high-status individuals. Indeed, low-status group members have been described as more conscious of stigmatization [18,19] and more accepting of being targeted by negative stereotypes [20]. These members also appeared to be more vulnerable to identity-threats, such as sexism [21] or their numerical underrepresentation (the solo status, [22]), as well as more attentive and responsive to threatening environmental cues [23]. These identity-threats consecutively impaired their performance [24], [25]. Additionally, it has been highlighted that status concerns can directly affect stereotype threat effects [26]. A greater decrease in performance was found among individuals dealing with a negative stereotype compared to those who did not while the non-targeted participants performed better than usual. It therefore seems plausible to think that simply being part of a low-status group could lead to performance differences when confronted with a stereotypical situation. We aimed to extend this new research line by considering two other aspects. First, as stereotype threat relies on social identity [27], we aimed to investigate whether the reported effect is restricted to gender only, as in [17], or if it is instead driven by a low chronic social status that leads individuals being more susceptible toward stereotypes in situational contexts. Indeed, gender has been repeatedly used in studies on social status, and gender differences have been intertwined with status differences in the literature [28], [10]. Some scholars even discussed gender differences as stemming from status inequality ([10], line 27). Hence, we investigated if we could observe different stereotype susceptibility across other social groups that differ on social status. We used height as an indicator of status for two reasons. First, height seems to be associated with a higher social status among men. Various studies have notably reported a positive association between height and educational achievement [29], [30]. Likewise, an overall positive link between earnings and height has been observed in blue-collar, clerical, or professional-technical professions [31], [32]. Second, discrimination based on height has been suggested as more threatening and relevant to men [33], [34]. Tall men tend to be more listened and respected, and they tend to enjoy better careers compared to short men [35]. In a similar vein, it has been revealed that tall presidents were more likely to be preferred, re-elected, and remembered, compared to short presidents [36], [37]. The preference for height in high-status occupations has also been recently observed in the media, for instance, president Sarkozy has been repeatedly mocked for his shortness [38], [39]. Based on our reasoning, we predicted that we would find greater stereotype susceptibility among short participants than tall participants, especially for men. However, if stereotype susceptibility was driven only by gender and not by status, as we claim, women should also be more affected compared to men in such situation. Second, we investigated whether age can increase the vulnerability of low-status group members. Children as young as 5 years old can be affected by stereotypes [40], and stereotype susceptibility effects are strengthened during childhood and adolescence [41]. However, the chronic asymmetry of gender status is rapidly learned during the childhood [42], [43]. Therefore, we aimed to replicate greater stereotype susceptibility of women in a first Experiment while Experiment 2 aimed to investigate the age effect on stereotype susceptibility to test whether older women could be more affected compared to younger ones. Experiment 3 will aim at replicating greater stereotype susceptibility of low-status group members using height as an indicator of chronic status.

To proceed, we evoked a stereotype to manipulate the situational asymmetry of the groups while retaining their chronic status (i.e., testing the "pure effects" of stereotyping, [17]). Because we believe that chronic low-status increases group members’ susceptibility to situational information, we hypothesize that they should be affected by both a positive and a negative stereotype. Conversely, individuals enjoying chronic high-status should be less responsive to both positive and negative stereotypes; therefore, high-status individuals should be less affected by the stereotype manipulation compared to members of a low-status group who perceive the status quo as a threat [25], [44].

### Overview of the studies

Participants engaged in specific tasks that do not activate a stereotype associated with the relevant groups in the experiments. We controlled the representation of the tasks and used only group-neutral tasks [45]. We further invoked stereotypes to rigorously test the extent to which chronic status and situational status affect stereotype susceptibility. This procedure notably allowed us to investigate how high-status individuals perform when they are confronted with a negative stereotype (i.e., when they keep their high chronic social status but endorse a low situational one).

## Experiment 1

### Method

#### Ethics Statement

Studies did not involve medical or health related experimentation. Studies 2 and 3 were conducted at the University of Lausanne or at the Swiss Federal Institute of Technology in Lausanne (Study 1), Switzerland.

Experimenters followed the APA Ethical Guidelines for Research (http://www.sandplay.org/pdf/APA_Ethical_Guidelines_for_Research.pdf). Participants were informed that the study consisted of performing a quick task and answering a few questions, and they were entitled to decline or withdraw from participation. Following the completion of the questionnaire, participants were debriefed and invited to ask any question about the research. The questionnaire simply asked them to indicate their age and sex. However, we added a measure of gender identification for Experiment 2 using a single item (e.g. “is it important for you to be a boy / a girl?”). They answered on a scale ranging from not at all (1) to very important (5). At the time of the three studies (2010–2013), no approval was needed in Switzerland to conduct research on human subjects. As stated by the Federal Administration of the Swiss Confederation (http://www.bag.admin.ch/themen/medizin/00701/00702/07558/index.html?lang=fr), the law relating to research on human subjects (i.e., constitutional article n°118b) came into effect in January 1^st^ 2014. Given this legislation, the present research project was not submitted to a research ethics board. Responses were completely kept confidential: Confidentiality of research records was strictly maintained by assigning all the provided data a code number. In addition, all participants have been orally debriefed, and particular attention was paid to ensure that participants understood the nature and the aim of the manipulation.

Participants were recruited in the hallway of the university in Study 1. Neither course credits nor payments were offered in exchange for participation. The participation was purely voluntary, and only verbal consent was obtained.

The data of Study 2 was collected during three open-days of the University of Lausanne. The aim of these open-days is to raise awareness of children and lay people on psychological research. Children came with their school during the first two days. Each school had to pre-register to join the study and parents had to sign an authorization to allow their children to participate. The third day was an open-day organized for lay people and children came with their parents. Two rooms were set for the study. Parents, teachers, and children who had yet to participate were waiting in a first room with some collaborators while the researchers and research assistants conducted the study with children, in a second room. Small individual boxes were set to prevent them from seeing or hearing the other participants. At the end, all participants were debriefed in the first room.

Data of Study 3 was collected during a first-year social psychology lecture in the psychology department for the university sample and during a psychology lecture in two Swiss high schools for the high school sample (Piccard and Chamblandes). Neither course credits (for students) nor payment was offered in exchange for participation. Again, the participation was purely voluntary, and only verbal consent was obtained.

#### Participants

One hundred and nineteen adults from a medium-size Swiss university volunteered in this experiment. Two men (one in the control condition with 28 hits and one in the men superiority condition with 23 hits) and 4 women (3 in the men superiority condition, with respectively 0, 1 and 21 hits and one in the control condition with 18 hits) had to be removed from the analysis because of too large Cook’s distance [46]. The analyses were conducted with 50 women and 63 men (*M*_age_ = 20.89 years, *SD* = 3.44). The distribution of the number of hits did not follow a normal curve (potentially because a representative part of the participants made zero hits). We addressed this issue by following the recommendation of Erceg-Hurn and Mirosevich [47] and ran the analyses with a robust estimator of variance. As this is true for the other experiments, this information will not be repeated.

#### Procedure and materials

**Fig 1:**
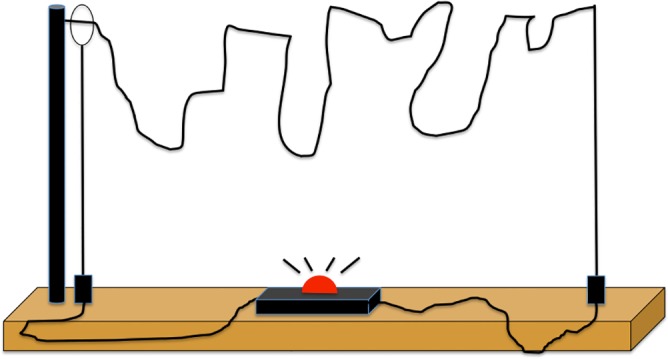
Illustration of the apparatus used in Experiment 1 and 2. The goal of the task was to move from start to finish touching the rod as little as possible.

Participants were randomly assigned to one of three experimental conditions. The first condition was a control condition and did not refer to gender (17 men, 17 women). We built a small apparatus for measuring fine motor skills for this experiment (see Fig 1 for an illustration). It included a wooden platform, a plastic wand, a metal washer combination, and a bent metal rod providing a winding track. The metal rod, the wand, and the washer combination were linked to a battery that made up an electric conductivity circuit. A loud buzzing sound and a red light alerted participants when the washer touched the rod, which indicated a hit. Fine motor skill tasks generally lead men and women performing equally well (see [48] for a review or [49] for a task similar than ours).

The goal of the task was to move from start to finish touching the rod as little as possible (control condition). In two other conditions, we experimentally evoked gender stereotypes. In the men superiority condition (23 men, 14 women), we indicated that usually “men succeed more on this task than do women”, which is a commonly evoked condition in the literature [50]. In the female superiority condition (23 men, 19 women), the same instruction was used except the words *men* and *women* were reversed to create a threatening stereotype for men. An experimenter (either a man or a woman) stood behind each participant, counted the number of hits, and tracked time (*M* = 36.06 sec, *SD* = 15.67). Before leaving, participants were asked to indicate their age and gender and repeat the instructions. All participants were able to recall the instructions correctly. They were then thanked and debriefed.

### Results

No relation was found between time and performance (*r* = -.10, *p* = .60). Likewise, experimenter gender had no effect on performance as a function of participant gender and condition or on the interaction between these two factors, as all *p*s >.10. As this finding is also true for Experiment 2, this information will not be repeated.

Performance in the control condition did not differ as a function of gender, *F*(1, 107) = 2.71, *p* = .10. Our prediction was tested by the interaction between gender (women coded as -0.5; men coded as 0.5) and a planned contrast opposing the men superiority condition (1) and the women superiority condition (-1) with the control condition set as (0), following the approach proposed by Judd, McClelland and Ryan [51]. The proper use of contrast analysis requires the planned contrast testing the hypothesis to be significant and the orthogonal contrast testing the residual to be non-significant. The orthogonal contrast therefore tested the control condition (2) against both the men superiority (-1) and the women superiority (-1) conditions.

**Fig 2:**
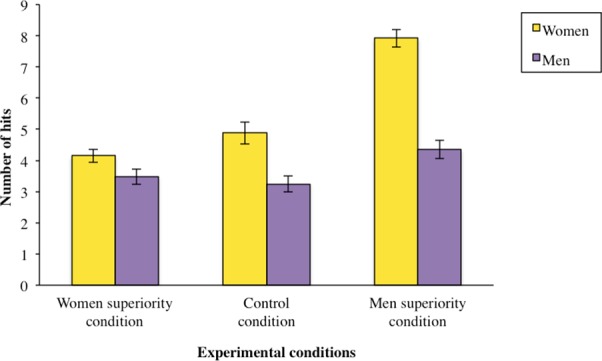
High Social Status protects men against Stereotype Susceptibility in a gender-neutral task (Experiment 1). Performance of women (represented in yellow bars) and men (represented in purple bars) in the respective three conditions: control condition, with no reference to gender; women superiority condition, positive for women and negative for men: ‘‘women succeed more on this task than do men”, and men superiority condition, negative for women and positive for men: ‘‘men succeed more on this task than do women”. Error bars are based on Standard Error of the mean.

The expected interaction between gender and the planned contrast (labeled as stereotype) reached significance, *F*(1,107) = 4.97, *p* = .028, η^2^_*p*_ = .04 (see Fig 2) whereas the interaction between gender and the orthogonal contrast did not, *F*(1,107) <1, *p* = .68 An analysis of simple effects revealed a significant effect of stereotype on women in a stereotype-consistent way, *F*(1, 107) = 15.52, *p* < .001, η^2^_*p*_ = .12. Women had significantly fewer hits in the women superiority condition (*M* = 4.15, *SD* = 2.45) than in the men superiority condition (*M* = 7.92, *SD* = 2.92), with the control condition falling in between (*M* = 4.88, *SD* = 3.01). This analysis did not reach significance for men, *F* <1, *p* = .32. We also observed a significant difference as a function of gender in the men superiority condition, *F*(1, 107) = 11.97, *p* < .001, η^2^_*p*_ = .10. This analysis did not reach significance in the women superiority condition, *F*(1, 107) <1, *p* = .39, η^2^_*p*_ = .005.

### Discussion

The results of this first experiment supported the hypothesis, which proposed that low-status group members should exhibit greater stereotype susceptibility compared to high-status group members. Women appeared to be more affected by stereotypes compared to men, as in [17]. Experiment 2 aimed to replicate this effect and to identify the role of age in the stereotype susceptibility of women.

## Experiment 2

### Method

#### Participants

The experiment took place at a medium-size Swiss university. Three hundred children voluntarily participated during open-days (113 girls and 187 boys). Their ages ranged from 7 to 15 years old (*M* = 11.37 years; *SD* = 1.63).

#### Procedure and materials

The procedure, materials and task were identical to Experiment 1. We added a measure of gender identification using a single item at the very end however (e.g. “is it important for you to be a boy / a girl?”). Participants answered on a scale ranging from not at all (1) to very important (5), *M* = 3.69, *SD* = 0.97. All participants were able to recall the instructions correctly. They were then thanked and debriefed. On average, participants spent 46.77 sec running on the track (*SD* = 18.05). The boys' superiority condition comprised 60 boys and 38 girls while the girls' superiority condition comprised 71 boys and 40 girls. The control condition consisted of 56 boys and 35 girls.

### Results

**Fig 3:**
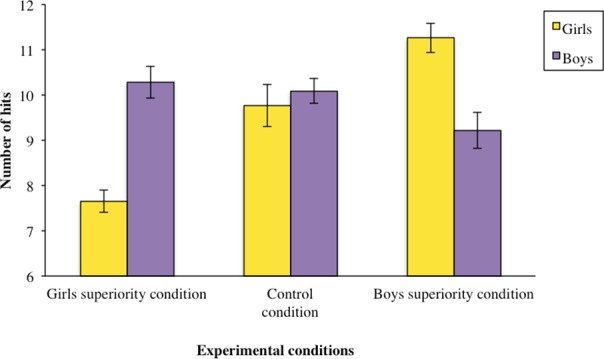
High Social Status protects boys against Stereotype Susceptibility in a gender-neutral task (Experiment 2). Performance of girls (represented in yellow bars) and boys (represented in purple bars) in the respective three conditions: control condition, with no reference to gender; girls superiority condition, positive for girls and negative for boys: ‘‘girls succeed more on this task than do boys”, and boys superiority condition, negative for girls and positive for boys: ‘‘boys succeed more on this task than do girls”. Error bars are based on Standard Error of the mean.

We again found no relation between time and performance (*r* = 0.07, *p* >.20). Performance in the control condition also did not differ as a function of gender, *F* < 1, *p* > .80. The interaction of interest was tested with the same planned and orthogonal contrasts as for Experiment 1. Interaction between gender and the planned contrast again yielded to a significant effect, *F*(1, 294) = 7.23, *p* = .007, η^2^_*p*_ = .024 (see Fig 3) whereas the interaction between the orthogonal contrast and gender did not, *F*(1, 294) <1, *p* = .98. An analysis of the simple effects revealed a significant effect of stereotype on girls in a stereotype-consistent way, *F*(1, 294) = 6.15, *p* = .014, η^2^_*p*_ = .020. Girls indeed made significantly fewer hits in the girls' superiority condition (*M* = 7.65, *SD* = 4.17) than in the boys' superiority condition (*M* = 11.26, *SD* = 8.04), with the control condition falling in the middle (*M* = 9.77, *SD* = 5.51). This analysis did not reach significance for boys, *F*(1, 294) = 1.27, *p* = .26. We also observed a significant difference as a function of gender in the women superiority condition, *F*(1, 294) = 7.23, *p* = .007, η^2^_*p*_ = .024. This test did not yield to any significant effect in the men superiority condition, *F*(1, 294) = 2.10, *p* = .15, η^2^_*p*_ = .007. No other effects reached significance, as all *p*s > .10.

Preliminary analyses indicated that the number of hits decreased with age, *b* = - 1.10, *F*(1, 288) = 26.63, *p* < .001. However, age did not interact with stereotype and gender or with the interaction between these two factors, all *p*s > .05. The same results emerged when testing a quadratic effect of age on the interaction of interest, *p*>.05. Likewise, interactions between the stereotype, gender and gender identification, between gender identification and gender and between the stereotype and gender identification did not yield to any significant effects; all *F*s were <1, *p*s >.45. It is important to note that the interaction between stereotype and gender remains significant when gender identification and its interactions are included in the model however, *F* (1, 288) = 7.61, *p* = .006, η^2^_*p*_ = .025.

### Discussion

The results of these first two experiments highlighted the greater stereotype susceptibility of women compared to men. Experiment 2 revealed that threatening situations affect girls more compared to boys as early as age 7 and that age does not seem to amplify this effect. Experiment 3 aimed to extend this result on another hierarchical social group. As a reminder, because height is more threatening to men compared to women, we postulated a double interaction in this experiment. Short men should be more affected by stereotypes than would tall men, whereas a threat associated with height would not affect women.

## Experiment 3

### Method

#### Participants

Two hundred and twenty-nine adult participants volunteered in this experiment. The data were collected in two steps. In the first step, we recruited university students as participants (*N* = 150) and, in the second step, we recruited high school students (*N* = 99). As no difference emerged between the two samples in height or performance (all *p*s > .13), the data were merged into a single database to have a better representation of gender and height in each experimental group. Two women identified as short (one in the short superiority condition with 30 hits and the other in the tall superiority condition with 31 hits) and two men (one identified as short who made 22 hits in the tall superiority condition and another identified as tall who made 15 hits in the short superiority condition) had to be removed from the analysis because of too large Cook’s distance [46]. The analysis was therefore conducted on 122 women and 123 men. Their average age was 20.53 years (*SD* = 4.20).

#### Procedure and materials

Upon arrival, participants were asked to indicate whether they perceived themselves to be short or tall (further named as height categorization). Sixty-eight women (34 in each condition) and 50 men (31 in the short condition and 19 in the tall condition) categorized themselves as short (*M*_height_ = 174.6cm, *SD* = 8.38 for men; *M*_height_ = 162.63cm, *SD* = 4.8 for women). Fifty-four women (35 in the short condition and 19 in the tall condition) and 73 men (39 in the short condition and 34 in the tall condition) categorized themselves as tall (*M*_height_ = 180.28cm, *SD* = 6.32 for men; *M*_height_ = 169.47cm, *SD* = 6.23 for women). They were then asked to remove their shoes to be measured (objective height) and were randomly assigned to the experimental conditions. Two experimental conditions were created. In the short superiority condition, we induced a positive stereotype by telling short individuals that usually, “short individuals succeed better on this task than do tall ones”. In the tall superiority condition, the instructions were the same but the words *short* and *tall* were reversed.

We used a balance task for this experiment. Participants were asked to stand on one leg touching the floor as little as possible. They were free to choose their preferred leg but were not allowed to change the leg after they started. The one-leg stance is included in classical clinical balance measures, such as the Berg Balance Scale [52]. To our knowledge, this ability is unrelated to height. Likewise, pretesting the university sample did not yield to any significant difference in performance as a function of objective height, *F* (1, 149) <1, *p* = .779.

The task consisted of two steps. First, participants had to stand on one leg for 15 seconds to measure their postural stability [53] and ensure their understanding of the task. Afterwards, they could start the task whenever they wanted. The timer started when they lifted their leg. The dependent variable was the number of times they touched the floor (referred to as "hits") in two minutes. An experimenter (either a man or a woman) counted the number of hits and tracked the time of the task. Before leaving, participants were asked to indicate their age and gender and to repeat the instructions. All participants were able to recall the instructions. They were then thanked and debriefed.

### Results

**Fig 4:**
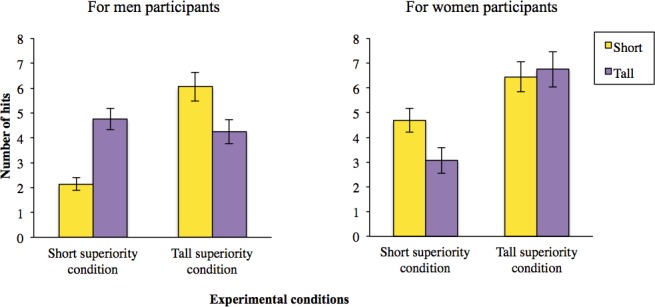
Women are protected against stereotypes on Height; Tallness protects men against Stereotype Susceptibility in a neutral task (Experiment 3). Performance of individuals who self-categorized as short (represented in yellow bars) and tall (represented in purple bars) in the respective two conditions: tall superiority condition, negative for short individuals and positive for tall ones: ‘‘tall individuals succeed better on this task than do short ones”, and short superiority condition, positive for short individuals and negative for tall ones: ‘‘short individuals succeed more on this task than do tall ones”. Error bars are based on Standard Error of the mean.

ANOVAs were conducted on the number of hits (the lower the number, the better the performance). We found a significant interaction among conditions, gender, and height categorization, *F*(1, 237) = 5.24, *p* = .023, η^2^_*p*_ = .021 (see Fig 4). A decomposition of this interaction indicated a significant interaction between conditions and height categorization for men, *F*(1, 237) = 6.60, *p* = .011, η^2^_*p*_ = .027, such that short men performed significantly worse in the tall superiority condition (*M* = 6.36, *SD* = 5.41) compared to the short superiority condition (*M* = 2.80, *SD* = 3.07). However, tall men did not perform differently across conditions, *F*<1, *p* > .50. On average, tall men made 4.23 hits in the tall superiority condition (*SD* = 4.47) and 4.94 (*SD* = 3.75) in the short superiority condition. Additionally, the condition by height interaction did not have any significant effect on women, *F*(1, 237) <1, *p >* .30. The interaction between height and conditions was also non-significant, *F*(1, 237) <1, *p >* .33.

The same analyses conducted with objective height as a predictor instead of height categorization did not have any significant effect, *F*(1, 237) <1, *p* > .50. Finally, we did not observe any significant effect within conditions as a function of objective height on men. We respectively found *F*(1, 237) = 3.27, *p* = .072, η^2^_*p*_ = .013 in the short superiority condition and *F*(1, 237) = 2.28, *p* = .13, η^2^_*p*_ = .009 in the tall superiority condition.

## General Discussion

The present paper focused on the role of chronic social status on stereotype susceptibility. We postulated that stereotypes would affect low-status group members more compared to high-status group members. Experiments 1 and 2 tested this hypothesis by comparing men, who endorse a high chronic status, to women, who endorse a low chronic status. Experiment 3 sought to replicate the observed differences with height (related with a chronic social status among men) to critically examine the role of gender and status in stereotype vulnerability. The interaction between height categorization and gender on performance provided support in favor of greater stereotype susceptibility of short men compared to tall men. We did not find such an effect on women. Data thus provided support for our main hypothesis.

Whereas no gender differences emerged in the control condition, the evoked stereotypes significantly affected women' (Experiment 1) and girls' performance (Experiment 2). Conversely, men' and boys' performance were more immune to change. In other words, the chronic high status of men protected them against a stereotype, which claimed that their situational status is lower compared to that of women. Of importance, this difference was found in children as young as 7 years of age. During their socialization, children learn about gender representations and gender discrimination [54]. As they age, they associate gender with domains [55], [56] and rapidly realize that men enjoy a higher social status than women. These gender representations can impair pupils’ performance [40], as it appears that threatening situations can differentially affect very young children. Experiment 3 extended this result to another intergroup comparison by considering height. In line with the above-mentioned literature, the finding revealed that self-categorized short men (individuals with a low chronic status) suffered more from identity-threats than did self-categorized tall men (individuals with a high chronic status) and that height affected men more than it did women. Individuals are susceptible to identity-threats as a function of the social comparison (i.e., the chronic status at play), since a high-status provides protection whereas low-status weakens individual performance when one has to deal with a salient negative stereotype.

Over the last two decades, scientific and political authorities in the Western world have joined their efforts to reduce the negative effects of stereotypes as much as possible and to promote equality across low and high-status groups [57], [58]. Despite this necessary effort to modify gender stereotypes, recent research has emphasized that these efforts have proved less effective than expected [59] and that gender gaps in performance could stem from factors other than gender stereotypes [60]. Our findings suggest that group comparisons could affect performance of individuals who suffer from a chronic low-status and that achieving gender neutrality may not necessarily lead to gender equality.

The research findings, which indicated that acting on the identity salience could diminish the effect of stereotype threat on performance, support this idea. For instance, identifying multiple identities before a mathematical task led to increased performance among women in a stereotype threat condition [61]. Individuation (i.e., focusing on an individual level rather than a group identity level) and self-affirmation (i.e. reminding characteristics and values important for the self) can protect individuals from being affected by a negative stereotype as well [62], [63]. All these manipulations decreased the salience of the low chronic status of women; therefore, they were less vulnerable to stereotype threat. Future interventions should therefore try to reduce group comparison in addition to working on attenuating the content of stereotypes.

This research could also inspire future research aiming at reducing stereotype threat effects. For instance, considering intelligence as incremental (i.e. as something we can develop across time) as opposed to fixed (i.e. as something inherent) proved effective in doing this [64], [65]. However, if it is true that it narrowed the usually observed performance gap between the stigmatized individuals and the non-targeted ones, the effect of the manipulation was stronger on low-status individuals than for high-status ones in both researches.

Our results could likewise be linked to the recent literature on gender educational achievements. Despite a similar gender achievement in mathematics under normal circumstances [66], [67] and considering the findings that show women as academically superior to men [68], the gender gap in favor of men seems to persist when considering national assessments [3]. It seems plausible to think that women could suffer from the asymmetry that results from the stereotype in such evaluative situations (i.e., by their low situational status on such tasks) whereas this is less likely to happen in more usual situations.

Four limitations need to be addressed. We highlighted, in line with our reasoning, that difference in performance emerged as a function of how individuals categorized themselves as tall or short and that objective height did not yield to any significant difference. Future research could however be interested in investigating if this holds true in other countries (especially in ones where women’ are taller than our participants, such as Germany or Sweden for example). Second, we did not address the psychological mechanism at play in our experiments. We suggest that belonging to a chronic high-status group offers a situational protection towards negative intergroup comparison, as people are aware of their social advantage compared to the out-group (e.g. men vs. women, tall men vs. short men). On the flipside, members of low-status group devote more attention to situations that either reminds them of such low status, or buffer their usual social disadvantage. It is therefore plausible to think that our results could be mediated by how individuals cope with such situational threats (such as the extent to which they think negatively during the task, [69]) and moderated by how identified they are towards the social identity at play [15]. Another limitation concerns the fact that we used gender as proxy of the social status regarding the multiple differences related to gender. Future research could therefore extend our reasoning to different other hierarchical groups. Fourth, this paper has focused solely on performance to highlight the greater susceptibility of low-status group members. However, research in economics has repeatedly emphasized important gender differences on competitiveness (see Niederle & Vesterlund for a review, [70]). Men are generally more likely to engage in competition, even when both gender perform equally well [71], [72]. In fact, we observed that most of these studies used stereotypically masculine tasks (such as a math task, see [73], [74], [75]). Our results suggest that such gap could be due to greater susceptibility of women in stereotypical situations rather than gender per se. Consequently, women should be as competitive than men in a more neutral situation; other hierarchical social groups could also potentially vary on competitiveness.

Notwithstanding these limitations, the present results support the hypothesis according to which individuals could be more or less susceptible to stereotypes as a function of their chronic social statuses. After several decades of research on stereotypes, the present paper suggests that future studies on stereotypes should consider status and group asymmetries to better understand why individuals can still underperform in evaluative situations, despite the recent progress in the attainment of better equality in the workforce.
